# Supplementary material for: Rapid deep learning-assisted predictive diagnostics for point-of-care testing
Source: Nat Commun. 2024 Feb 24;15:1695. doi: 10.1038/s41467-024-46069-2 (PMC10894262; doi:10.1038/s41467-024-46069-2)
Supplement: Supplementary file 1 — Supplementary Information [file 41467_2024_46069_MOESM1_ESM.pdf]

Supplementary Information

Rapid Deep Learning-Assisted Predictive Diagnostics for Point-of-Care Testing

Seungmin Lee[1,2*], Jeong Soo Park[1,3 *], Hyowon Woo[1*], Yong Kyoung Yoo[4], Dongho Lee[5], Seok Chung[3], Dae Sung Yoon[2,6,7], Ki-Baek Lee[1], and Jeong Hoon Lee[1,5†]

[1] *Department of Electrical Engineering, Kwangwoon University, 20 Kwangwoon-ro, Nowon, Seoul 01897, Republic of Korea*

[2] *School of Biomedical Engineering, Korea University, 145 Anam-ro, Seongbuk, Seoul 02841, Republic of Korea*

[3]*School of Mechanical Engineering, Korea University, 145 Anam-ro, Seoungbuk-gu, Seoul 02841, Republic of Korea*

[4]*Department of Electronic Engineering, Catholic Kwandong University, 24, Beomil-ro 579 beon-gil, Gangneung-si, Gangwon-do 25601, Republic of Korea*

[5]*CALTH Inc., Changeop-ro 54, Seongnam, Gyeonggi 13449, Republic of Korea*

[6]*Interdisciplinary Program in Precision Public Health, Korea University, Seoul 02841, Republic of Korea*

[7]*Astrion Inc, Seoul 02841, Republic of Korea*

* These authors contributed equally.

† Corresponding authors: jhlee@kw.ac.kr

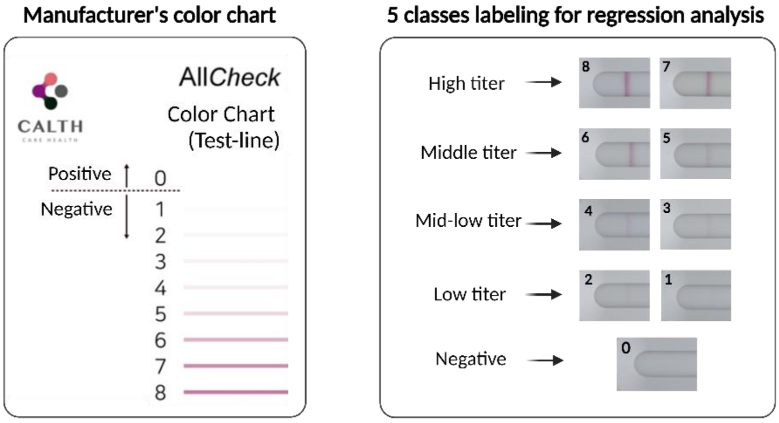

Supplementary Fig. 1. Images of the manufacturer-supplied color chart and corresponding categorized images.

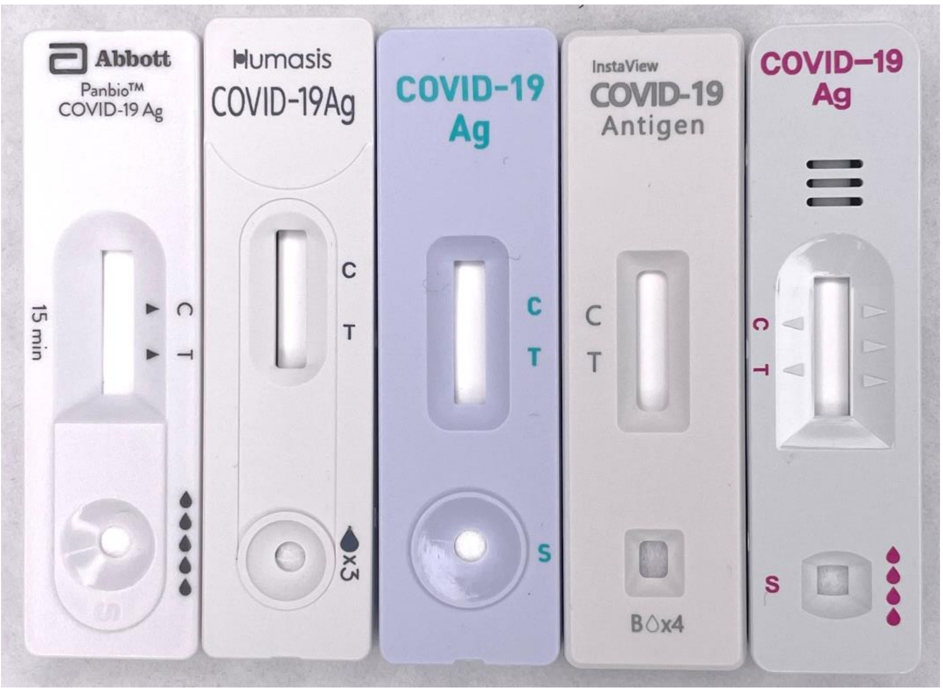

Supplementary Fig. 2. Images of various commercial kits with different form factors. From left to right: Panbio COVID-19 Ag (Abbott, USA), COVID-19 Ag Test (Humasis, Republic of Korea), GENEDIA COVID-19 (GCMS, Republic of Korea), InstaView COVID-19 (SGmedical, Republic of Korea), COVID-19 Ag (GenBody, Republic of Korea).

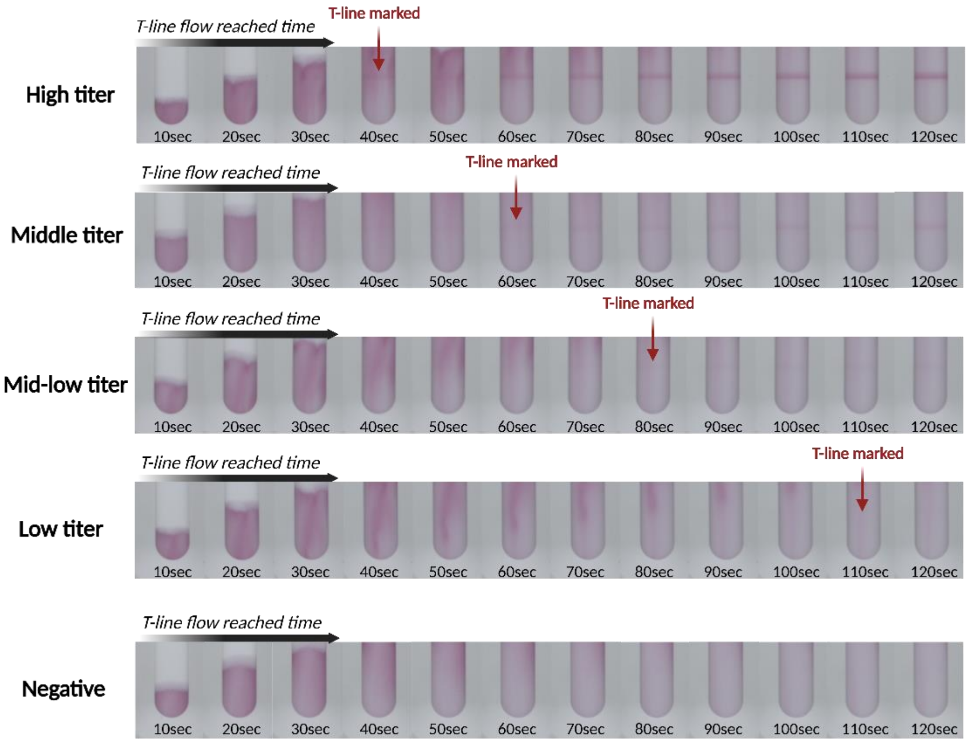

Supplementary Fig. 3. Time-Series LFA Images. Sequential images depicting the progression of the assay over time.

Supplementary Table 1. Comparison of performance metrics in published literature, including method, assay time, cost, and equipment.

Target	Product (corp.)	method	Assay time	Cost	Additional reader equipment	Procedure
COVID-19	(Grant, Anderson et al. 2020) [1]	LFA	20 min	NA		Simple
	(Liu, Zhan et al. 2021) [2]	LFA	30 min	NA		Simple
	(Chen, Liu et al. 2022) [3]	immunoassay	20 min	NA	Required	Simple
	(Klumpp-Thomas, Kalish et al. 2021) [4]	ELISA	3~5 hr	NA	Required	Complex
	(Dao Thi, Herbst et al. 2020) [5]	RT-LAMP	>35 min	NA	Required	Intermediate
	(Bokelmann, Nickel et al. 2021) [6]	RT-LAMP	>35 min	NA		Intermediate
	(Patchsung, Jantarug et al. 2020) [7]	CRISPR	2 hr	NA	Required	Complex
	(Adnan, Khandker et al. 2022) [8]	PCR	2~3 hr	NA	Required	Complex
	(Song, Hong et al. 2023) [9]	PCR (microfluidic)	30 min	NA	Required	Simple
	(Cheong, Yu et al. 2020) [10]	PCR (nanoplasmonic)	17 min	NA	Required	Simple
	(Najjar, Rainbow et al. 2022) [11]	Electrochemical sensor (microfluidic)	1 hour	13 $	Required	Simple
Influenza A	(Wu, Yuan et al. 2016) [12]	LFA	15 min	NA		Simple
	(Lee, Chu et al. 2021) [13]	RT-LAMP	60 min	NA		Intermediate
	(Leirs, Tewari Kumar et al. 2016) [14]	ELISA	3~5 hr	NA	Required	Complex
hCG	(Kim, Yoo et al. 2017) [15]	LFA	30 min	NA		Simple
	(Ahi, Torul et al. 2022) [16]	SERS (microfluidic)	50 min	NA	Required	Complex
	(Wang, Li et al. 2018) [17]	ELISA	>3 hr	NA	Required	Complex
cardiac troponin I (cTnI)	(Uddin, Bhuiyan et al. 2021) [18]	ELISA (microfluidic)	45 min	NA		Simple
C-Reactive Protein (hsCRP)	(Ballard, Joung et al. 2020) [19]	Immunoassay + DL	< 12min	NA	Required	Intermediate
Sepsis	(Zupančič, Jolly et al. 2021) [20]	Electrochemical sensor	50 min	NA	Required	Intermediate

Supplementary table 2. Comparison of performance metrics in commercialized products, including method, assay time, cost, and equipment.

Target	Product (corp.)	method	Assay time	Cost	Additional reader equipment	Procedure
COVID-19	Ellume	Rapid kit (fluoresence)	<15 min	25 $		Simple
	BinaxNOW COVID-19 (Abbott)	Rapid kit	<15 min	7 $		Simple
	Rapid antigen test (Roche)	Rapid kit	<15 min	9 $		Simple
	ID NOW COVID-19 (Abbott)	RT-LAMP	<12 min	40 $	Required	Simple
	Allplex™ SARS-CoV-2 (Seegene)	PCR	2~3 hr	1200 $	Required	Complex
	DiaPlexQ™ Novel Coronavirus (SolGent)	PCR	2~3 hr	1000 $	Required	Complex
	COVID-19 S-Protein ELISA kit (Abcam)	ELISA	3~5 hr	975 $	Required	Complex
	SARS-CoV-2 N Detection Kit (Sino Biological)	ELISA	3~5 hr	700 $	Required	Complex
Influenza A/B	BINAXNOW influenza A+B card (Abbott)	Rapid kit	<15 min	29 $		Simple
	ID NOW influenza A&B (Abbott)	RT-LAMP	<13 min	70 $	Required	Simple
	PowerChek™ Influenza Virus (Kogene)	PCR	2~3 hr	NA	Required	Complex
	Human Influenza A Virus ELISA Kit (Abbexa)	ELISA	3~5 hr	572 $	Required	Complex
hCG	Quickvue hCG combo (Quidel)	Rapid kit	5 min	6 $		Simple
	Clearview hCG Tests (Abbott)	Rapid kit	5 min	6 $		Simple
	β-HCG Rapid Test Kit (Vitrosens)	Rapid kit	<15 min	NA		Simple
	Human hCG ELISA Kit (Abcam)	ELISA	3~5 hr	730 $	Required	Complex

Supplementary Table 3. Training and test datasets for each experiment.

Target		Train		Test
		Initial training set	Additional training set	
**Initial training set (COVID-19)**	Positive	380	0	54
	Negative	214	0	30
**5 different models (COVID-19)**	Positive	380	200	400
	Negative	214	100	200
**TIMESAVER (Clinical test of COVID-19)**	Positive	380	50	156
	Negative	214	50	96
**Influenza**	Positive	380	32	128
	Negative	214	16	64
**Troponin I**	Positive	380	16	64
	Negative	214	8	32
**hCG (for expert)**	Positive	380	20	40
	Negative	214	10	20
**hCG (self-tests)**	Positive	380	16	64
	Negative	214	8	30

Supplementary Table 4. Information of clinical samples (P: patient, N: normal, NP/OP: Nasopharyngeal and oropharyngeal)

Sample number	Sample collections	Variants	Sex (0: female 1: male)	Ages	Ct values
**P#1**	Saliva	Omicron	0	53	20.65
**P#2**	Saliva	Omicron	1	44	22.2
**P#3**	Saliva	Omicron	0	25	22.22
**P#4**	Saliva	Omicron	0	53	22.52
**P#5**	Saliva	Omicron	0	33	22.84
**P#6**	Saliva	Omicron	1	38	26.84
**P#7**	Saliva	Omicron	0	53	26.84
**P#8**	Saliva	Omicron	0	73	28.12
**P#9**	Saliva	Omicron	1	64	28.25
**P#10**	Saliva	Omicron	0	62	30.65
**P#11**	Saliva	Omicron	1	48	31.22
**P#12**	Saliva	Omicron	0	57	31.72
**N#1**	Saliva	-	1	26	N
**N#2**	Saliva	-	1	28	N
**N#3**	Saliva	-	0	26	N
**N#4**	Saliva	-	1	57	N
**N#5**	Saliva	-	1	49	N
**N#6**	Saliva	-	1	38	N
**N#7**	Saliva	-	0	31	N
**N#8**	Saliva	-	1	29	N
**N#9**	Saliva	-	1	36	N
**N#10**	Saliva	-	0	58	N
**N#11**	Saliva	-	0	42	N
**N#12**	Saliva	-	0	23	N

Sample number	Sample collections	Variants	Sex (0: female 1: male)	Ages	Ct values
**P#1**	NP/OP	Omicron	-	-	16.74
**P#2**	NP/OP	Omicron	-	-	16.91
**P#3**	NP/OP	Omicron	-	-	17.57
**P#4**	NP/OP	Omicron	0	62	17.8
**P#5**	NP/OP	Omicron	-	-	17.8
**P#6**	NP/OP	Omicron	1	65	17.81
**P#7**	NP/OP	Omicron	-	-	18.04
**P#8**	NP/OP	Omicron	1	44	18.72
**P#9**	NP/OP	Delta	0	55	19.2
**P#10**	NP/OP	Omicron	-	-	19.41
**P#11**	NP/OP	Omicron	0	58	20.24
**P#12**	NP/OP	Delta	0	42	20.9
**P#13**	NP/OP	Omicron	1	65	20.94
**P#14**	NP/OP	Omicron	-	-	21.44
**P#15**	NP/OP	Omicron	0	65	21.84
**P#16**	NP/OP	Omicron	0	25	22.22
**P#17**	NP/OP	Omicron	1	41	22.32
**P#18**	NP/OP	Omicron	0	29	22.32
**P#19**	NP/OP	Omicron	0	39	22.7
**P#20**	NP/OP	Omicron	0	33	22.84
**P#21**	NP/OP	Omicron	0	36	22.84
**P#22**	NP/OP	Omicron	1	83	23.48
**P#23**	NP/OP	Omicron	0	54	23.49
**P#24**	NP/OP	Delta	0	35	23.7
**P#25**	NP/OP	Omicron	1	55	23.9
**P#26**	NP/OP	Delta	0	63	24.1
**P#27**	NP/OP	Delta	1	59	24.9
**P#28**	NP/OP	Omicron	1	46	25.69
**P#29**	NP/OP	Omicron	1	58	26.5
**P#30**	NP/OP	Omicron	1	46	26.78
**P#31**	NP/OP	Omicron	1	22	26.78
**P#32**	NP/OP	Omicron	0	53	26.84
**P#33**	NP/OP	Omicron	1	38	26.84
**P#34**	NP/OP	Delta	0	19	28
**P#35**	NP/OP	Omicron	0	78	28.12
**P#36**	NP/OP	Omicron	1	64	28.25

**P#37**	NP/OP	Delta	1	19	29
**P#38**	NP/OP	Delta	1	18	29
**P#39**	NP/OP	Delta	1	18	31
**P#40**	NP/OP	Delta	1	16	32
**N#1**	NP/OP	-	1	23	N
**N#2**	NP/OP	-	1	23	N
**N#3**	NP/OP	-	0	24	N
**N#4**	NP/OP	-	0	26	N
**N#5**	NP/OP	-	1	26	N
**N#6**	NP/OP	-	1	27	N
**N#7**	NP/OP	-	1	27	N
**N#8**	NP/OP	-	1	31	N
**N#9**	NP/OP	-	0	31	N
**N#10**	NP/OP	-	1	33	N
**N#11**	NP/OP	-	1	36	N
**N#12**	NP/OP	-	1	38	N
**N#13**	NP/OP	-	0	40	N
**N#14**	NP/OP	-	1	47	N
**N#15**	NP/OP	-	1	48	N
**N#16**	NP/OP	-	0	53	N
**N#17**	NP/OP	-	0	58	N
**N#18**	NP/OP	-	1	58	N
**N#19**	NP/OP	-	0	62	N
**N#20**	NP/OP	-	0	68	N

Supplementary Table 5. Clinical Sample Titer Labels for 5 Classes (P: Patient, N: Normal, NP/OP: Nasopharyngeal and Oropharyngeal)

Sample number	Sample collections	Ct values	Label	Titer
**P#1**	Saliva	20.65	10-12	High
**P#2**	Saliva	22.2	10-12	High
**P#3**	Saliva	22.22	8-9	Middle
**P#4**	Saliva	22.52	8-9	Middle
**P#5**	Saliva	22.84	8-9	Middle
**P#6**	Saliva	26.84	8-9	Middle
**P#7**	Saliva	26.84	6-7	Mid-low
**P#8**	Saliva	28.12	6-7	Mid-low
**P#9**	Saliva	28.25	4-5	Low
**P#10**	Saliva	30.65	4-5	Low
**P#11**	Saliva	31.22	4-5	Low
**P#12**	Saliva	31.72	4-5	Low
**N#1**	Saliva	N	0-3	Negative
**N#2**	Saliva	N	0-3	Negative
**N#3**	Saliva	N	0-3	Negative
**N#4**	Saliva	N	0-3	Negative
**N#5**	Saliva	N	0-3	Negative
**N#6**	Saliva	N	0-3	Negative
**N#7**	Saliva	N	0-3	Negative
**N#8**	Saliva	N	0-3	Negative
**N#9**	Saliva	N	0-3	Negative
**N#10**	Saliva	N	0-3	Negative
**N#11**	Saliva	N	0-3	Negative
**N#12**	Saliva	N	0-3	Negative

Sample number	Sample collections	Variants	Sex (0: female 1: male)	Ages
**P#1**	NP/OP	16.74	10-12	High
**P#2**	NP/OP	16.91	10-12	High
**P#3**	NP/OP	17.57	10-12	High
**P#4**	NP/OP	17.8	10-12	High
**P#5**	NP/OP	17.8	10-12	High
**P#6**	NP/OP	17.81	10-12	High
**P#7**	NP/OP	18.04	10-12	High
**P#8**	NP/OP	18.72	10-12	High
**P#9**	NP/OP	19.2	10-12	High
**P#10**	NP/OP	19.41	8-9	Middle
**P#11**	NP/OP	20.24	8-9	Middle
**P#12**	NP/OP	20.9	8-9	Middle
**P#13**	NP/OP	20.94	8-9	Middle
**P#14**	NP/OP	21.44	8-9	Middle
**P#15**	NP/OP	21.84	8-9	Middle
**P#16**	NP/OP	22.22	8-9	Middle
**P#17**	NP/OP	22.32	8-9	Middle
**P#18**	NP/OP	22.32	8-9	Middle
**P#19**	NP/OP	22.7	8-9	Middle
**P#20**	NP/OP	22.84	8-9	Middle
**P#21**	NP/OP	22.84	6-7	Mid-low
**P#22**	NP/OP	23.48	6-7	Mid-low
**P#23**	NP/OP	23.49	6-7	Mid-low
**P#24**	NP/OP	23.7	6-7	Mid-low
**P#25**	NP/OP	23.9	6-7	Mid-low
**P#26**	NP/OP	24.1	6-7	Mid-low
**P#27**	NP/OP	24.9	6-7	Mid-low
**P#28**	NP/OP	25.69	6-7	Mid-low
**P#29**	NP/OP	26.5	6-7	Mid-low
**P#30**	NP/OP	26.78	6-7	Mid-low
**P#31**	NP/OP	26.78	6-7	Mid-low
**P#32**	NP/OP	26.84	4-5	Low
**P#33**	NP/OP	26.84	4-5	Low
**P#34**	NP/OP	28	4-5	Low
**P#35**	NP/OP	28.12	4-5	Low
**P#36**	NP/OP	28.25	4-5	Low

**P#37**	NP/OP	29	4-5	Low
**P#38**	NP/OP	29	4-5	Low
**P#39**	NP/OP	31	4-5	Low
**P#40**	NP/OP	32	4-5	Low
**N#1**	NP/OP	N	0-3	Negative
**N#2**	NP/OP	N	0-3	Negative
**N#3**	NP/OP	N	0-3	Negative
**N#4**	NP/OP	N	0-3	Negative
**N#5**	NP/OP	N	0-3	Negative
**N#6**	NP/OP	N	0-3	Negative
**N#7**	NP/OP	N	0-3	Negative
**N#8**	NP/OP	N	0-3	Negative
**N#9**	NP/OP	N	0-3	Negative
**N#10**	NP/OP	N	0-3	Negative
**N#11**	NP/OP	N	0-3	Negative
**N#12**	NP/OP	N	0-3	Negative
**N#13**	NP/OP	N	0-3	Negative
**N#14**	NP/OP	N	0-3	Negative
**N#15**	NP/OP	N	0-3	Negative
**N#16**	NP/OP	N	0-3	Negative
**N#17**	NP/OP	N	0-3	Negative
**N#18**	NP/OP	N	0-3	Negative
**N#19**	NP/OP	N	0-3	Negative
**N#20**	NP/OP	N	0-3	Negative

Supplementary References

1. Grant, B.D., et al., *SARS-CoV-2 coronavirus nucleocapsid antigen-detecting half-strip lateral flow assay toward the development of point of care tests using commercially available reagents.* Analytical chemistry, 2020. **92**(16): p. 11305-11309.
2. Liu, Y., et al., *fM–aM Detection of the SARS-CoV-2 Antigen by Advanced Lateral Flow Immunoassay Based on Gold Nanospheres.* ACS Applied Nano Materials, 2021. **4**(12): p. 13826-13837.
3. Chen, Y., F. Liu, and L.P. Lee, *Quantitative and ultrasensitive in situ immunoassay technology for SARS-CoV-2 detection in saliva.* Science Advances, 2022. **8**(21): p. eabn3481.
4. Klumpp-Thomas, C., et al., *Standardization of ELISA protocols for serosurveys of the SARS-CoV-2 pandemic using clinical and at-home blood sampling.* Nature Communications, 2021. **12**(1): p. 113.
5. Dao Thi, V.L., et al., *A colorimetric RT-LAMP assay and LAMP-sequencing for detecting SARS-CoV-2 RNA in clinical samples.* Science Translational Medicine, 2020. **12**(556): p. eabc7075.
6. Bokelmann, L., et al., *Point-of-care bulk testing for SARS-CoV-2 by combining hybridization capture with improved colorimetric LAMP.* Nature Communications, 2021. **12**(1): p. 1467.
7. Patchsung, M., et al., *Clinical validation of a Cas13-based assay for the detection of SARS-CoV-2 RNA.* Nature Biomedical Engineering, 2020. **4**(12): p. 1140-1149.
8. Adnan, N., et al., *Detection of SARS-CoV-2 by antigen ELISA test is highly swayed by viral load and sample storage condition.* Expert Review of Anti-infective Therapy, 2022. **20**(3): p. 473-481.
9. Song, M., S. Hong, and L.P. Lee, *Multiplexed Ultrasensitive Sample-to-Answer RT-LAMP Chip for the Identification of SARS-CoV-2 and Influenza Viruses.* Advanced Materials, 2023. **35**(10): p. 2207138.
10. Cheong, J., et al., *Fast detection of SARS-CoV-2 RNA via the integration of plasmonic thermocycling and fluorescence detection in a portable device.* Nature Biomedical Engineering, 2020. **4**(12): p. 1159-1167.
11. Najjar, D., et al., *A lab-on-a-chip for the concurrent electrochemical detection of SARS-CoV-2 RNA and anti-SARS-CoV-2 antibodies in saliva and plasma.* Nature Biomedical Engineering, 2022. **6**(8): p. 968-978.
12. Wu, F., et al., *Multiplexed detection of influenza A virus subtype H5 and H9 via quantum dot-based immunoassay.* Biosensors and Bioelectronics, 2016. **77**: p. 464-470.
13. Lee, D., C.-H. Chu, and A.F. Sarioglu, *Point-of-Care Toolkit for Multiplex Molecular Diagnosis of SARS-CoV-2 and Influenza A and B Viruses.* ACS Sensors, 2021. **6**(9): p. 3204-3213.
14. Leirs, K., et al., *Bioassay Development for Ultrasensitive Detection of Influenza A Nucleoprotein Using Digital ELISA.* Analytical Chemistry, 2016. **88**(17): p. 8450-8458.
15. Kim, C., et al., *Battery operated preconcentration-assisted lateral flow assay.* Lab on a Chip, 2017. **17**(14): p. 2451-2458.
16. Ahi, E.E., et al., *A capillary driven microfluidic chip for SERS based hCG detection.* Biosensors and Bioelectronics, 2022. **195**: p. 113660.
17. Wang, W., et al., *Ultrasensitive ELISA for the detection of hCG based on assembled gold nanoparticles induced by functional polyamidoamine dendrimers.* Analytica Chimica Acta, 2018. **1042**: p. 116-124.

18. Uddin, M.J., N.H. Bhuiyan, and J.S. Shim, *Fully integrated rapid microfluidic device translated from conventional 96-well ELISA kit.* Scientific Reports, 2021. **11**(1): p. 1986.
19. Ballard, Z.S., et al., *Deep learning-enabled point-of-care sensing using multiplexed paper-based sensors.* npj Digital Medicine, 2020. **3**(1): p. 66.
20. Zupančič, U., et al., *Graphene Enabled Low-Noise Surface Chemistry for Multiplexed Sepsis Biomarker Detection in Whole Blood.* Advanced Functional Materials, 2021. **31**(16): p. 2010638.
