# Supplementary material for: Rapid deep learning-assisted predictive diagnostics for point-of-care testing
Source: Nat Commun. 2024 Feb 24;15:1695. doi: 10.1038/s41467-024-46069-2 (PMC10894262; doi:10.1038/s41467-024-46069-2)
Supplement: Supplementary file 3 — Description of Additional Supplementary Files [file 41467_2024_46069_MOESM3_ESM.pdf]

<u>Description of Additional Supplementary Files</u>

Supplementary Movie Legend

Supplementary Movie 1: The demonstration of the developed algorithm for rapid predictive diagnostics (TIMESAVER).
