# Supplementary material for: Rapid deep learning-assisted predictive diagnostics for point-of-care testing
Source: Nat Commun. 2024 Feb 24;15:1695. doi: 10.1038/s41467-024-46069-2 (PMC10894262; doi:10.1038/s41467-024-46069-2)
Supplement: Supplementary file 5 — Reporting Summary [file 41467_2024_46069_MOESM5_ESM.pdf]

nature portfolio

Corresponding author(s): Jeong Hoon Lee

Last updated by author(s): Jan 31, 2024

Reporting Summary

Nature Portfolio wishes to improve the reproducibility of the work that we publish. This form provides structure for consistency and transparency in reporting. For further information on Nature Portfolio policies, see our Editorial Policies and the Editorial Policy Checklist.

Statistics

For all statistical analyses, confirm that the following items are present in the figure legend, table legend, main text, or Methods section.

n/a	Confirmed	
☐	☒	The exact sample size (*n*) for each experimental group/condition, given as a discrete number and unit of measurement
☐	☒	A statement on whether measurements were taken from distinct samples or whether the same sample was measured repeatedly
☒	☐	The statistical test(s) used AND whether they are one- or two-sided<br>*Only common tests should be described solely by name; describe more complex techniques in the Methods section.*
☒	☐	A description of all covariates tested
☒	☐	A description of any assumptions or corrections, such as tests of normality and adjustment for multiple comparisons
☐	☒	A full description of the statistical parameters including central tendency (e.g. means) or other basic estimates (e.g. regression coefficient) AND variation (e.g. standard deviation) or associated estimates of uncertainty (e.g. confidence intervals)
☒	☐	For null hypothesis testing, the test statistic (e.g. *F*, *t*, *r*) with confidence intervals, effect sizes, degrees of freedom and *P* value noted<br>*Give P values as exact values whenever suitable.*
☒	☐	For Bayesian analysis, information on the choice of priors and Markov chain Monte Carlo settings
☒	☐	For hierarchical and complex designs, identification of the appropriate level for tests and full reporting of outcomes
☒	☐	Estimates of effect sizes (e.g. Cohen's *d*, Pearson's *r*), indicating how they were calculated

Our web collection on statistics for biologists contains articles on many of the points above.

Software and code

Policy information about availability of computer code

Data collection	Images were taken from built-in camera software using LabVIEW v2019 SP1 (National Instruments Co., USA).<br>Images were systematically captured at 10-second intervals across varying time periods (0.5, 1, 2, 3, and 4 minutes).
Data analysis	The overall source codes used in this study is available at: (https://github.com/Artinto/Rapid_Deep_Learning-Assisted_Predictive_Diagnostics_for_Point-of-Care_Testing) which is archived in https://zenodo.org/records/10582339 [https://doi.org/10.5281/zenodo.10582339].<br>Deep learning models were trained on Ubuntu 16.04 with python 3.9.0 and pytorch(torch==2.0.1) as a backend.<br>The well-known architectures (YOLOv3, DenseNet-121, ResNet-18, ResNet-34, ResNet-50, GRU, LSTM) were used in this study.<br>Data were analyzed using Microsoft Excel and Graphpad Prism v 8.0.<br>Biorender(webpage), Adobe Photoshop v 2020, and Adobe Illustrator v 2020 software were used for graphical analyses.

For manuscripts utilizing custom algorithms or software that are central to the research but not yet described in published literature, software must be made available to editors and reviewers. We strongly encourage code deposition in a community repository (e.g. GitHub). See the Nature Portfolio guidelines for submitting code & software for further information.

Data

Policy information about availability of data

All manuscripts must include a data availability statement. This statement should provide the following information, where applicable:

- Accession codes, unique identifiers, or web links for publicly available datasets
- A description of any restrictions on data availability
- For clinical datasets or third party data, please ensure that the statement adheres to our policy

Source data are provided with this paper. Example images used in this study are available at https://zenodo.org/records/10582232 [https://doi.org/10.5281/zenodo.10582232]. For tuning ResNet-50, we employed IMAGENET1k (https://www.kaggle.com/c/imagenet-object-localization-challenge/overview).

Research involving human participants, their data, or biological material

Policy information about studies with human participants or human data. See also policy information about sex, gender (identity/presentation), and sexual orientation and race, ethnicity and racism.

Reporting on sex and gender	Clinical information of patients (i.e. age) was considered as a risk factor for the susceptibility or prognosis of SARS-CoV-2 infection. However, it does not affect the result of the study for development and optimization of the diagnostic method using positive samples. Gender was not considered.
Reporting on race, ethnicity, or other socially relevant groupings	Respiratory samples were systematically gathered from individuals diagnosed with COVID-19 infection at Seoul St. Mary's Hospital (Republic of Korea). Race and ethnicity were not considered.
Population characteristics	The sample of patients and normal was spanned ages 16 to 83 and balanced sex distribution.
Recruitment	Respiratory samples were systematically gathered from individuals diagnosed with COVID-19 infection at Seoul St. Mary's Hospital between April 2021 and May 2022. The study received approval from the institutional review board at Seoul St. Mary's Hospital, and participants provided informed consent. Respiratory samples were systematically gathered from individuals diagnosed with COVID-19 infection at Seoul St. Mary's Hospital between April 2021 and May 2022. The study received approval from the institutional review board at Seoul St. Mary's Hospital, and participants provided informed consent.
Ethics oversight	Seoul St. Mary’s Hospital, with the approval of the Institutional Review Board Committee (KC21TIDI0134K).

Note that full information on the approval of the study protocol must also be provided in the manuscript.

Field-specific reporting

Please select the one below that is the best fit for your research. If you are not sure, read the appropriate sections before making your selection.

☒ Life sciences ☐ Behavioural & social sciences ☐ Ecological, evolutionary & environmental sciences

For a reference copy of the document with all sections, see nature.com/documents/nr-reporting-summary-flat.pdf

Life sciences study design

All studies must disclose on these points even when the disclosure is negative.

Sample size	The algorithm trained with standard and clinical sample images (n=13,404) from . It was tested with 1,229 data from various targets, including COVID-19, influenza, and hCG. Clinical sample images (n=252, positive: 156, and negative: 96) for blind-test. Clinical sample images were taken from COVID-19 patients (n=52) and healthy controls (n=32).<br>Sample size calculation is not required because it is a research design that has been experimented by collecting samples prospectively.
Data exclusions	Some LFA kits showed false positives. In this case, we excluded the images.
Replication	All experiment were independently implemented and repeated at least more than three times. All attempts at replication were successful. In the case of an experiment with an algorithm, the same result was shown when the model parameters were fixed.
Randomization	Our experiment is not about comparing the effects of the two drugs or diagnostic methods in different randomly assigned groups but about comparing and verifying two different diagnostic methods in diagnosing the same samples which are prospectively collected and anonymized. As a result, randomization is not applicable. Our study was taken with patients and normals who were verified through PCR.<br>The training dataset and the test dataset were prepared with random generation.
Blinding	Untrained individual, LFA experts, and the suggested algorithm outcome were blinded to labels (positive/negative).

Reporting for specific materials, systems and methods

We require information from authors about some types of materials, experimental systems and methods used in many studies. Here, indicate whether each material, system or method listed is relevant to your study. If you are not sure if a list item applies to your research, read the appropriate section before selecting a response.

Materials & experimental systems

n/a	Involved in the study
☒	☐ Antibodies
☒	☐ Eukaryotic cell lines
☒	☐ Palaeontology and archaeology
☒	☐ Animals and other organisms
☒	☐ Clinical data
☒	☐ Dual use research of concern
☒	☐ Plants

Methods

n/a	Involved in the study
☒	☐ ChIP-seq
☒	☐ Flow cytometry
☒	☐ MRI-based neuroimaging
